# Impact of COVID-19 pandemic on hip fractures: the central London experience COVID-related urgent geriatric hip trauma (COUGH) study COVERT ( COVid Emergency-Related Trauma and orthopaedics) collaborative

**DOI:** 10.1007/s11845-021-02687-z

**Published:** 2021-06-28

**Authors:** Chang Park, Kapil Sugand, Arash Aframian, Catrin Morgan, Nadia Pakroo, Charles Gibbons, Michael Fertleman, Dinesh Nathwani, Rajarshi Bhattacharya, Khaled M. Sarraf

**Affiliations:** 1 Imperial College Healthcare NHS Trust, London, UK; 2 Chelsea & Westminster NHS Foundation Trust, London, UK

**Keywords:** Best practice tariff, COVID-19, Epidemiology, Hip fracture, Mortality, National hip fracture database, Neck of femur fracture, Surgery

## Abstract

**Introduction:**

COVID-19 has been recognized as the unprecedented global health crisis in modern times. The purpose of this study was to assess the impact of COVID-19 on treatment of neck of femur fractures (NOFF) against the current guidelines and meeting best practice key performance indicators (KPIs) according to the National Hip Fracture Database (NHFD) in two large central London hospitals.

**Materials and methods:**

A multi-center, longitudinal, retrospective, observational study of NOFF patients was performed for the first ‘golden’ month following the lockdown measures introduced in mid-March 2020. This was compared to the same time period in 2019.

**Results:**

A total of 78 cases were observed. NOFFs accounted for 11% more of all acute referrals during the COVID era. There were fewer overall breaches in KPIs in time to theatre in 2020 and also for those awaiting an orthogeriatric review. Time to discharge from the trust during the pandemic was improved by 54% (p < 0.00001) but patients were 51% less likely to return to their usual residence (p = 0.007). The odds ratio was significantly higher for consultant surgeon-led operations and consultant orthogeriatric-led review in the post-COVID era. There was no significant difference in using aerosol-generating anaesthetic procedures or immortality rates between both years.

**Conclusion:**

The impact of COVID-19 pandemic has not adversely affected the KPIs for the treatment of NOFF patients with significant improvement in numerous care domains. These findings may represent the efforts to ensure that these vulnerable patients are treated promptly to minimize their risks from the coronavirus.

## Introduction

### Consequences of COVID-19 on neck of femur fractures: the British response

The morbidity and mortality of COVID-19 have been particularly significant in the elderly over 70 years of age [1]. This vulnerable group has been urged to take more stringent precautions compared to the general population and to stay at home from mid-March for at least 3 months [2, 3]. Despite this advice, neck of femur fractures (NOFFs) continue to occur in the vulnerable demographic as the majority tend to occur within their home environment (despite the reduction in the NOFFs occurring outdoors). Hence, the prevalence is unlikely to be significantly affected by the social distancing measures imposed unlike other injuries that saw a reduction during the pandemic [4–6].

### Guidelines for NOFF

National Institute for Health and Care Excellence (NICE) [7] sets out the gold standard guidelines for NOFF treatment in the UK. The guidelines have been shown to improve the morbidity and mortality associated with these injuries [5, 8, 9]. The management of such fractures is audited nationally by the means of the National Hip Fracture Database (NHFD). Trusts delivering care are financially incentivized to meet the key performance indicators (KPI) set out within the guidelines, and the data is published via the NHFD [10] on a quarterly basis.

### Involvement of surgical bodies

The British Orthopaedic Association [11] has recently raised the concern on how to best allocate surgical resources if the COVID-19 pandemic overwhelms the NHS, and the potential need to make difficult moral and ethical choices in extreme cases. The professional body has suggested the use of the Swansea Hip interrogation Fracture Tool [11] to assist in the triage and prioritization of NOFF patients for surgery. The guidance suggested that a prolonged reduction of operative capacity of below 20% should trigger this new process of triaging.

## Aim

A multi-centred, parallel, longitudinal, retrospective, observational study was conducted to evaluate the impact of the COVID-19 pandemic on NOFF in central London (known as a COVID hotspot) for 5 weeks prior to and during the COVID-19 pandemic (i.e. 2019 vs 2020).

## Materials and methods

### Patient sampling

Sampling of all acute trauma referrals and operative caseload was performed at two large central London hospitals consisting of one level 1 trauma center (i.e. major trauma center) and a level 2 trauma unit (e.g. district general hospital). Those patients with a NOFF were identified, and data from the NHFD were assessed.

### Study period

The study period was from the start of social distancing on 17 March 2020 to 20 April 2020. This period also included the morning following more firm ‘lockdown’ measures on 24 March 2020. This was compared to the same 5-week interval in March–April 2019 prior to any COVID-19 related measures to compare its impact.

### Data points

Data from the NHFD were examined including for its completeness and attainment of best practice tariff (BPT) criteria. The Nottingham Hip Fracture Score (NHFS) was calculated alongside the Clinical Frailty Score (CFS), and as a sum of both, the SHiFT score. The COVID-19 status for patients in 2020 was recorded including the time and results of testing. Thirty-day mortality outcomes were collected.

### Statistical analysis

All the data were recorded using Microsoft Excel (Microsoft, Washington, USA) as well as being verified by 3 authors for its accuracy. The data were treated non-parametrically which was confirmed with a Kolmogorov–Smirnov test. The median (± median absolute deviation) were calculated and supported by Mann–Whitney U test for p-values, set at a statistical significance of p < 0.05. Risk (or prevalence) and odds ratios (with 95% confidence intervals) were calculated as well as their significance using Fisher’s test for p-values, again set at p < 0.05.

### Human and animal rights

This study only represents retrospective data from patient notes, and no experimentation was conducted on either humans or animals. All human and animal rights were adhered to throughout the entire study.

## Results

The results have been tabulated in Table 1.

**Table 1:** Results from both pre- and post-COVID study periods

	Pre-COVID		COVID		p
	45		33		
Patient demographics	*n* =	%	*n* =	%	
Median age	81		84		0.4
Male	10	22	10	30	
Female	35	78	23	70	
Records complete	44	98	22	67	
Meets best practice tariffs	23	51	15	45	
**Residence**					
Own/Sheltered	42	93	28	85	
Residential	1	2	1	3	
Nursing home	2	4	4	12	
**Admission and assessment**				0	
Admitted via ED	42	93	33	100	
Fall on ward as inpatient	3	7	0	0	
Median time taken to be admitted to orthopaedic ward (hours)	7		8.7		0.02
Nerve block	33	73	17	52	
Pre-op Hb	128.5		124		0.96
**Hip fracture laterality**					
Left	26	58	14	42	
Right	19	42	19	58	
**Pre-fracture mobility**					
Freely mobilize without aids	16	36	11	33	
Mobile outdoor with one aid	12	27	7	21	
Mobile outside with two aids or frame	8	18	5	15	
Some indoor mobility but never goes outside without help	9	20	10	30	
**Prognostic indicators**					
Delirium assessment	41	91	26	79	
Delirium assessment—alertness	0		0		
Delirium assessment—AMT4	0		1		
Delirium assessment—attention	0		1		
Delirium assessment—fluctuation	0		0		
Delirium assessment score 4AT	0		2.5		
New pressure ulcer	0	0	2	6	
Specialist fall assessment	45	100	32	97	
Median AMTS	10		8		0.03
Median ASA	3		3		0.3
Nutritional status complete	45	100	30	91	
**Bone protection**					
Assessed—not for protection	19	42	19	58	
On no treatment—pending DEXA scan	19	42	6	18	
Started on admission	7	16	8	24	
**Surgery**					
**Type of fracture**					
Number of operations	45	100	32	97	
Intracapsular—undisplaced	1	2	1	3	
Intracapsular—displaced	22	49	12	38	
Trochanteric grade A1/A2	15	33	13	41	
Trochanteric grade A3	4	9	1	3	
Subtrochanteric	3	7	4	13	
Femoral Shaft Fracture	0	0	1	3	
Non-operative	0	0	1	3	
% pathological	0	0	0	0	
Median time to surgery (hours)	28.8		21.7		0.2
n breach (36 hours)	16	36	8	25	
**Type of operation**					
Arthroplasty—bipolar hemi cemented	15	33	11	34	
Arthroplasty THR Hybrid	5	11	1	3	
Internal fixation—IM nail (long)	7	16	8	25	
Internal fixation—IM nail (short)	5	11	2	6	
Internal fixation—sliding screw	11	24	9	28	
Internal fixation—cannulated screws	2	4	1	3	
**Type of anaesthetic**					
GA (AGP)	23	51	16	50	
Non AGP	22	49	16	50	
N = consultant surgeon	25	56	28	88	
n = consultant anaesthetist	45	100	32	100	
**Post-operative/treatment**					
n = assessed by physio	42	93	33	100	
n = out of bed D1	33	73	19	58	
Orthogeriatric review	45	100	32	97	
n = consultant orthogeriatric review	13	29	28	85	
Median time until orthogeriatric review (hours)	25.5		19.7		0.2
n breach (72 hours)	3	7	1	3	
Reoperation in 3 months	1	2	0	0	
Median time to discharge from ward (days)	10		5		0.0003
Median time to discharge from trust (days)	14		6.5		0.00001
Discharged to own/sheltered home	28	62	10	30	
Death	4	9	5	15	
**Scores**					
Median NHFS	5		5		0.2
Median CFS	5		6		0.3
Median SHiFT	10		11		0.0007

*NHFS* Nottingham Hip Fracture Score, *CFS* Clinical Fragility Score, *SHiFT* Swansea Hip interrogation Fracture Tool, *AMTS* abbreviated mental test score, *ED* Emergency Department, *Hb* Hemaglobin, *ASA* American Society of Anesthesiologists, *DEXA* dual energy X-ray absorptiometry, *THR* total hip replacement, *IM* intramedullary, *GA* general anesthesia, *AGP* aerosol-generating procedure, *D* day

### Pre-COVID era

For the pre-COVID period in 2019, there were 239 new referrals and 147 patients undergoing operative procedures in this time frame. Forty-five patients had a NOFF with data available on the NHFD. This represents 19% of all referrals.

### COVID era

For the COVID period in 2020, there were 110 referrals and 76 patients undergoing operative procedures. This represented a 64% reduction of referrals as compared to 2019 and halving of operative numbers. Thirty three of those patients had a NOFF and represents 30% of all acute referrals. The number of NOFF in 2020 was approximately a third less than in 2019. One patient had a recorded NOFF but refused to have surgery and was managed non-operatively, which was corroborated with a SHiFT score of 15 for the non-operative pathway.

#### SHiFT score

The SHiFT score was the only score that was statistically different between both cohorts, but the categorization remained the same. A score of 9–12 indicated an intention to operate which must be agreed upon a review by two consultant-led decision as well as the possibility of waiting 7 days for a second review to reconsider surgery. The median Nottingham Hip Fracture Score of 5 in both years indicated a mortality rate prediction of 9.8% which differed from the national 30-day mortality rate of circa 6.7% according to the British Orthopaedic Association [12].

#### Abbreviated mental test score (AMTS)

The AMTS is scored out of 10 and is a commonly used test to screen for possible underlying cognitive impairment. There was a significant difference in AMTS scores with the 2020 cohort being classified as mildly confused based on the median score of 8 compared to a score of 10 in 2019 (p = 0.03), indicating increasing vulnerability of those admitted during the COVID era.

### COVID status and mortality

A total of 39% (*n* = 13) of NOFF patients had a clinical suspicion of the virus on presentation, and 70% (n = 23) were tested for COVID-19 via PCR swab. Of these, 2 patients (6%) were positive for COVID-19. The swab results took a median of 48.2 hours to return, but only 30% of those results were available prior to surgery. Exactly 9% (*n* = 4) NOFF patients died within 30 days of injury in 2019 compared to 15% (*n* = 5) patients in 2020 during the COVID period. There was no statistical significance in mortality rate between both years. These results are depicted in Table 2.

**Table 2:** COVID results

	Yes	No
COVID suspected	13 (39%)	20 (61%)
COVID swab	23 (70%)	10 (30%)
Median date of COVID swab	08/04/2020	
Median time of COVID swab	14:00	
Median date of COVID result	10/04/2020	
Median time of COVID result	14:11	
COVID positive	2 Yes (6%)	
	21 No (64%)	
Results available pre-op	7 Yes (30%)	
	16 No (70%)	

### Prevalence (risk) and odds ratios

The risk ratio (RR), equivalent to prevalence ratio (PR) in this observational study, and the odds ratio (OR) alongside Fisher’s exact p-values were calculated and tabulated (Table 3). The post-COVID period (i.e. 2020) was compared to pre-COVID (i.e. 2019). Only statistically significant factors were included.

**Table 3:** Prevalence and odds ratios of years 2020 (post-COVID) vs 2019 (pre-COVID)

Factors	Prevalence and odds ratios		p-value (Fisher’s)
Records completed	*PR*	0.68	0.002
	*OR*	0.05	
Bone protection—on no treatment but pending DEXA scan	*PR*	0.43	0.03
	*OR*	0.30	
Consultant surgeon led-surgery	*PR*	1.53	0.007
	*OR*	4.48	
Consultant orthogeriatric review	*PR*	2.94	< 0.000001
	*OR*	13.8	
Discharge to own home/sheltered accommodation	*PR*	0.49	0.007
	*OR*	0.26	

### Median times for review and intervention along the patient journey

**Fig. 1:**
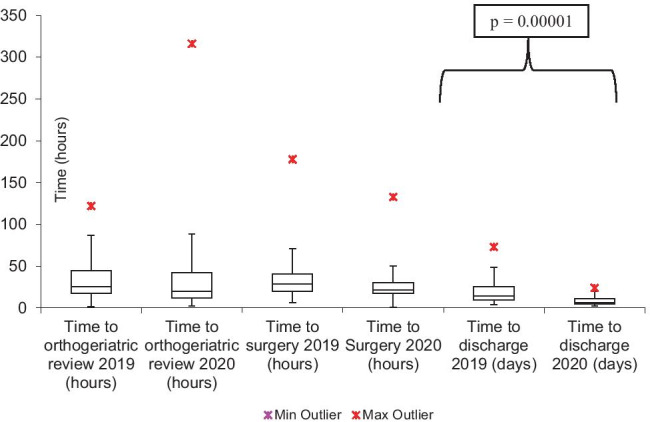
Box plots on timings of reviews and interventions compared between 1 year

The median timings for orthogeriatric review, time to surgery, and to discharge from hospital trust were depicted in Fig. 1. The only statistically significant difference was within the time to discharge from the hospital. Nevertheless, the comparison between both years suggests a reduced range and faster timings, during the COVID period compared to 2019. The least variability was seen in the median time to orthogeriatric review, but the differences were statistically insignificant, much like the time to surgery.

## Discussion

### NOFF prevalence and demographics

In 2020, there were 33 NOFF patients eligible for the NHFD database compared to 45 in 2019. Although an overall absolute reduction by a quarter, the percentage of referrals that were NOFF increased in 2020 to 30% of all referrals from 19% in the preceding year, representing a greater share of the departmental workload during the COVID era. As expected, there was no substantial difference in the NOFF demographics between years including the risk stratification scoring systems (NHFS and CFS). Furthermore, the median age and the gender split were similar, as was the ASA identical pre- and post-COVID further supporting the homogeneity of the cohorts. The majority of patients were admitted from their own home in both periods, and their pre-injury mobility, fracture configuration, and type of operative intervention were well matched between both periods.

### Impact of COVID on patient surgical pathway and treatment

The COVID-19 pandemic has necessitated the reconfiguration of resources and pathways to treat patients with COVID-19 and also to protect highly vulnerable patients and healthcare workers [13, 14]. Despite these radical changes, those with NOFF during the COVID period did not see a deterioration in their time to treatment. As per standard procedure, all NOFF patients in this study were still discussed in the daily trauma meeting in the presence of surgeons, anaesthetists, and orthogeriatricians who come together in a multidisciplinary approach to provide individualized and judicious evidence-based treatment.

Although there was a 24% increase in the time to admission from the emergency department to the orthopaedic ward (p = 0.02), this could reflect on the decline in the number of the allied healthcare professionals and hospital staff while also dealing with the concurrent pressure of COVID with an unprecedented demand of side rooms in any suspected COVID-19 patients. The time to theatre was not significantly changed in both periods (Fig. 1).

The SHiFT study [11] envisaged a scenario whereby they would require a tight triage of patients undergoing operative intervention for NOFF. Fortunately, this has not occurred, with a small but insignificant decrease in median times to theatres and number of breaches during the pandemic. This finding is likely due to the overall reduction of trauma workload found during this period leading to an overall decrease in operative demand. There has also been a recognition that these patients, often elderly with existing comorbidities, represent the most vulnerable to COVID-19, and therefore would be prioritized for operative intervention in a timely manner even in the midst of the pandemic. The more prompt the surgical intervention, the quicker patients would be able to recover and mobilize to reduce the risk of immobility-related complications including pneumonia.

### Surgical factors

The risk of COVID-19 transmission is increased in any aerosol-generating procedures (AGP), such as intubation, and there are stringent infection control measures for the anaesthetic process [15–18]. Surprisingly, the risk of AGP such as general anaesthetic with intubation was not reduced between both groups. The expectation would be to reduce the use of AGPs as much as possible to minimize the risks, but this has not been the case with 50% continuing to have AGP for the operative procedure, which has not significantly changed from 2019 (51%). This may represent the medical complexity of these NOFF patients and the limitation in the use of non-AGP anaesthetic such as the anticoagulation status of patients or accounting for the reduced baseline median AMTS in 2020 leading to reduced compliance during regional anaesthetic and the operation.

Whereas all operations were led by consultant anaesthetists, the odds of a consultant surgeon leading the operation during the COVID-19 period was increased by a factor of 4.48 in spite of an identical median ASA grade (ASA 3) between both years. This was due to redeployment of senior surgeons to the trauma theatre since all elective and private practice was compelled to cease, thus increasing their availability as the primary surgeon in the main trauma theatre.

### Quality of post-operative assessment

The multi-disciplinary approach to the treatment of NOFF patients is paramount. NOFF patients often have complex medical needs, and all should be reviewed by an orthogeriatrician within 72 hours and also assessed by a physiotherapist to encourage mobilization on day 1 post-op. This is to help facilitate earlier safe discharge from the acute site, more importantly during the pandemic to reduce the patient’s risk of contracting COVID-19.

A total of 97% of patients were reviewed by an orthogeriatric team member in 2020, and 85% of the reviews were at consultant level (p < 0.000001), compared to only 29% in 2019 representing the odds of a consultant-led orthogeriatric review was 13.8 times higher in the COVID-19 period. There was a reduction in the time taken to orthogeriatric review too, which improved by over 20% to under 20 hours. There was no statistical difference in the number of breaches over 72 hours. This represents a greater degree of senior-led care in the management of NOFF patients during the pandemic who were reviewed sooner to provide the gold-standard care to ensure swifter (but safe) discharge from the acute setting.

This care has meant that during the COVID-19 period, there has been a statistically significant improvement in the time to discharge from the trust to support this in 2020. This is comparable at 6.5 days in 2020 compared to 14 days in 2019. By minimizing the time in hospital, it is hoped that the chances of an adverse outcome due to COVID-19 is reduced.

### Mortality and morbidity

The recent COVID-Surg study has found a mortality of nearly 25% in patients undergoing operative intervention during the COVID period, with age over 70 years being a significant risk factor [19]. This places patients presenting with NOFF fractures during the COVID-19 pandemic into a high-risk stratification by default. Maniscalco et al. [20] found a mortality rate of 18% in NOFF patients within the Italian experience, with 82% of mortality associated with a positive COVID-19 status.

In our experience, the 30-day mortality of NOFF patients during the pandemic was 15% compared to 9% in 2019 and significantly greater than the mortality rate nationally at 6.7% published by the NHFD [10]. Traditionally, the 30-day mortality rate has been cited as high as 10% [12]. Of those who died in 2020, one patient died from a confirmed diagnosis of COVID-19. This patient was discharged home with a package of care 9 days following admission, but 6 days following discharge was readmitted with new respiratory symptoms and was confirmed PCR positive for COVID-19 on readmission. She died 11 days later with primary cause of death being COVID-19. Due to the known incubation period for COVID-19, it cannot be said for certain if this patient contracted COVID-19 during their inpatient stay or following discharge. Her operative intervention however was prompt, and her care met all the best practice tariffs with an intramedullary nailing being performed within 15 hours of diagnosis. In the worst-case scenario as suggested in the SHiFT study, this patient had a score of 10 which would have been triaged to potential surgery. Therefore, operative intervention may have been delayed by up to 7 days and this was unlikely to have improved her outcomes.

Another patient had a positive COVID PCR test whilst an inpatient, but this was 3 days after their operative intervention. They were discharged home on day 22 from admission following a subsequent negative COVID PCR test. Again, one can only speculate where this patient contracted COVID-19 (although was felt to be suffering with symptoms of COVID-19 on initial assessment), but this patient again did not breach their time to surgery, and despite a SHiFT score of 14, benefitted from their prompt time to theatres. Whilst this is only a snapshot across a 5-week period, it does warrant further study to investigate the ongoing impact of the pandemic on NOFF mortality, how this compares to the figures suggested by other studies and show a true national representation.

## Conclusion

This study represents a multi-centered experience of COVID-19 on the treatment of NOFF. The prevalence and demographics of NOFF remained unchanged during the COVID-19 period. NOFF represented an overall reduction of trauma referrals during the lockdown period but still consisted of a greater proportion of the departmental workload. The gold-standard care, as outlined by the KPIs of NHFD, of vulnerable NOFF patients did not suffer at the height of the pandemic indicating that patient safety was prioritized in the face of an unprecedented crisis. In fact, there was an enhanced level of care with respect to senior and consultant input as well as time to review and discharge from the hospital without a significant difference in mortality rate. In the absence of a cure or vaccine at the time, the continued focus of prioritization of these at-risk patients is recommended to avoid morbidity and mortality in the COVID-19 era.

## Data Availability

Available upon request.
